# Editorial: Impact of COVID-19 on cancer care and rehabilitation

**DOI:** 10.3389/fresc.2023.1257427

**Published:** 2023-07-31

**Authors:** Melanie Dalby, Shereen Nabhani-Gebara

**Affiliations:** ^1^ Department of Pharmacy, King’s College Hospital NHS Foundation Trust, London, United Kingdom; ^2^ Department of Pharmacy, Kingston University, London, United Kingdom

**Keywords:** cancer, techonology, COVID-19, pandemic (COVID19), oncology

**Editorial on the Research Topic** Impact of COVID-19 on cancer care and rehabilitation

The pandemic of COVID-19 had a significant impact on healthcare worldwide, affecting the care of many high risk patients including those with cancer (1, 2). Referrals for cancer from primary care reduced during the pandemic, many screening services were put on hold and treatments were drastically disrupted (1, 3). Healthcare teams across the world had to work hard to find innovative ways to manage the care of their cancer patients whilst keeping them safe.

For example, short-course radiotherapy was used to delay the need for surgery in locally advanced rectal cancer and reduced attendance requirement in the hospital setting (4). This has been shown to have benefits in both routine clinical practice and during the pandemic. A study comparing surgery one week and 4–8 weeks after short-course radiotherapy showed no difference in local recurrence, disease free survival and overall survival (5).

A number of healthcare settings utilised technology in their outpatient clinics switching to virtual consultations thus further reducing hospital attendance whilst still providing a high quality level of care (6). Studies have shown that virtual consultations are well received by patients and healthcare professionals if appropriate equipment and training are provided.

The British Oncology Pharmacy Association collated changes in oncology pharmacy services during the pandemic and provided a guide for its members on reviewing and evaluating these changes for consideration for future services in the endemic phase (3). An example of continued use after the pandemic was the use of telephone consultations and couriering out medication to patients at home to avoid visits to the hospital.

The purpose of this collection of papers is to showcase and share learning from those who have had experience of these changes including implementing the use of IT solutions and/or IT science to assist and improve clinical outcomes for patients.

There are four articles in this research topic each providing insight into the affects COVID-19 has had on cancer care around the world and the initiatives that have been implemented. Each study had a unique angle to investigate with some focusing on the impact of mental wellbeing on treatment delays while others examining predictive models measuring the impact of the pandemic on hospital admissions.

Cigarini et al. described the impact that the pandemic had on cancer care pathways in northern Italy and proposes a tool for providing high levels of quality care even in the most critical health situations.

He et al. described factors influencing delayed treatment in people living with breast cancer. This study took place in Chengdu, China and described a cross sectional investigation of close to 400 patients living with breast cancer.

Liu et al. report on an interrupted time series analysis investigating the impact of the pandemic on monthly hospital admissions for cancer patients in Henan, China. They also review the delays to diagnosis and recognise that telemedicine is not only a helpful IT solution for those that cannot visit a hospital but it can also be used to support reduction of the backlog caused by the pandemic.

The final article submitted by Jani et al. reviews the risk factors associated with worse outcomes from COVID-19 in cancer patients and understanding a suitable method to mitigate the risks of COVID-19 for this patient cohort.

The COVID-19 pandemic was disruptive to most health care services and healthcare professionals displayed resilience and agility in adapting the care they provided to their patients. The impact and resulting learnings were valuable and will help better prepare for any future similar disruptions.

Both editors of this research topic wish to thank all submitting authors for their work. As the editorial team, we hope that this research continues to prove a useful resource to provide care to cancer patients during unprecedented times.
